# Improving the adoption of a school-based nutrition program: findings from a collaborative network of randomised trials

**DOI:** 10.1186/s13012-025-01417-8

**Published:** 2025-01-16

**Authors:** Courtney Barnes, Rachel Sutherland, Lisa Janssen, Jannah Jones, Katie Robertson, Justine Gowland-Ella, Nicola Kerr, Aimee Mitchell, Karen Gillham, Alison L. Brown, Luke Wolfenden

**Affiliations:** 1 Hunter New England Population Health, Hunter New England Local Health District, Newcastle, NSW Australia; 2 School of Medicine and Public Health, University of Newcastle, Newcastle, NSW Australia; 3 Population Health Research Program, Hunter Medical Research Institute, Newcastle, NSW Australia; 4 National Centre of Implementation Science, University of Newcastle, Newcastle, NSW Australia; 5 Health Promotion Service, Central Coast Local Health District, Gosford, NSW Australia; 6 Health Promotion Service, Mid North Coast Local Health District, Coffs Harbour, NSW Australia

**Keywords:** Implementation, Adoption, School, Nutrition, Strategies

## Abstract

**Background:**

Public health nutrition interventions, including school-based programs, are a recommended approach to improve child dietary behaviours. However, the adoption of effective school-based nutrition programs face numerous challenges, including the limited evidence on effective strategies to maximise implementation and adoption of such programs. This study aimed to address this evidence gap by employing a novel collaborative network trial design to evaluate a series of implementation strategies employed by three NSW Local Health Districts, to improve school adoption of an effective school-based nutrition program (‘SWAP IT’).

**Methods:**

Three independent, two arm parallel group randomised controlled trials were conducted simultaneously to examine the potential effectiveness of implementation strategies on school adoption of SWAP IT. Schools were randomised to either a high intensity (various implementation strategies), or a business as usual (minimal support) group. Measures and data collection processes were harmonised across the three trials to provide individual school-level data for planned pooled analyses. The primary outcome was school adoption of SWAP IT, objectively measured via electronic registration records. Logistic regression analyses were used to assess school adoption of SWAP IT for each trial. Meta-analyses were also conducted to pool the effects of the three trials and allow the comparison of the potential relative effects of the different strategies.

**Results:**

A total of 287 schools were included in the study: Trial 1 (*n* = 164), Trial 2 (*n* = 64) and Trial 3 (*n* = 59). Relative to control, we found increased odds of adoption in Trial 1 that employed a combination of the educational materials and local facilitation strategies (OR 8.78; 95%CI 2.90, 26.56; *p* < 0.001), but no significant differences in adoption in Trial 2 or 3 that employed solely the educational materials strategy. Pooled data suggests the combination of educational materials and local facilitation has a greater effect on adoption compared to educational materials alone (OR 4.18; 95%CI 1.60, 10.04; *n* = 3 studies; indirect effect).

**Conclusion:**

Findings of this study indicate that local facilitation is an important strategy to increase school adoption of SWAP IT, and potentially other health promotion programs.

**Trial registration:**

The trials were prospectively registered with Australia New Zealand Clinical Trials Register:

- ANZCTR, ACTRN12622000257763, Registered 11/2/2022, https://www.anzctr.org.au/Trial/Registration/TrialReview.aspx?id=383515&isReview=true
- ANZCTR, ACTRN12622000406707, Registered 9/3/2022 https://www.anzctr.org.au/Trial/Registration/TrialReview.aspx?id=383701&isReview=true
- ANZCTR, ACTRN12622000252718, Registered on 11/2/2022, https://www.anzctr.org.au/Trial/Registration/TrialReview.aspx?id=383513&isReview=true

**Supplementary Information:**

The online version contains supplementary material available at 10.1186/s13012-025-01417-8.

Contributions to the literature

- This study addresses a crucial evidence gap, with little evidence currently available to guide the scale-up of effective school-based nutrition programs.
- Findings of the study provide insight into the potential effect of strategies that could be employed to scale-up school-based nutrition programs.
- This study utilises a novel approach to testing implementation strategies that enables the tailoring to local context.

## Background

Chronic diseases are the leading cause of death and disability globally, with much of this burden being preventable [1, 2]. Dietary risks are among the primary modifiable factors for chronic disease [2]. The implementation of public health nutrition interventions has been recommended to reduce the chronic disease burden, particularly those targeting the dietary behaviours of children. [3] Schools are a recommended setting for nutrition intervention, given the setting provides near universal access to children for prolonged periods [4–6], and children consume up to two-thirds of their daily energy intake during school hours [7]. Despite the existence of a range of effective school-based nutrition interventions, [8] few are integrated into usual school practice and delivered routinely to students, severely limiting the public health potential of such interventions.

An important impediment to the large-scale adoption of effective school nutrition initiatives is limited evidence of effective strategies to implement these initiatives at scale [9]. For example, a recent Cochrane review of implementation strategies for school-based health promotion programs found randomised trials testing strategies to implement nutrition policies of programs in schools accrue at a rate of less than one per year. Further, just one randomised controlled trial was included in the review where implementation of nutrition initiatives was sought ‘at scale’, defined by the authors as 50 or more schools [9]. Furthermore, strategies identified as effective in improving program implementation in a study undertaken in one jurisdiction, may not be effective in another, particularly if there are substantive differences in implementation barriers, implementation support or other contextual factors between jurisdictions. As such, more collaborative approaches to implementation research have been recommended to help advance the field and our understanding of what implementation strategies may work, and in which contexts [10].

The current research production process is insufficient to advance implementation science, or to guide timely action by agencies responsible for facilitating the adoption of health promotion programs in schools. The limited evidence-base may reflect a lack of academic funding available for implementation and dissemination research, and the challenges for academics in undertaking implementation trials which are often complex, time consuming, expensive, and require partnerships with health and other community organisations. [11] Innovations in methods are required to support more rapid generation and use of implementation research to improve health services. Methods of integrating research into the ‘usual business’ of health and other services; that leverage their resources, expertise and infrastructure; and that foster greater collaboration between services for collective improvement have been suggested as one means of doing so [10]. For example, an innovative ‘Master Protocol design’ (an adaptive ‘platform trial), involving methods harmonisation, was employed to simultaneously test the effects of multiple therapies for COVID-19. The research was embedded within the U.K National Health Service and established the effectiveness of a range of therapies in a fraction of the time of conventional clinical trial systems in the US [12, 13]. Similar approaches could facilitate generation and use of evidence to improve the implementation of nutrition initiatives in community settings such as schools.

Guidelines regarding a range of school-based policies and practices to reduce chronic disease risks have been developed across jurisdictions, the vast majority of which are remarkably consistent in their recommendations [3, 14–16]. As such, governments nationally and internationally have invested in supporting the adoption and implementation of these recommendations at the same time [17]. This convergence of activity provides a considerable opportunity for collaboration to study different strategies undertaken in different contexts for the implementation of the same prevention policies or practices. The co-ordination of such research activities, for example, through the harmonisation of key research methods, would also enable more direct comparisons of the effects of different implementation strategies. Collaborative efforts may also facilitate knowledge exchange, increase the availability of evidence to support local decision making and policy development and improve research translation across jurisdictions.

Health Promotion Units within the 15 Local Health Districts (LHDs) of New South Wales (NSW), Australia, are responsible for providing services to their community based on priorities set by the NSW Ministry of Health, and in response to needs identified by the local community. These services include supporting the implementation of health promotion programs in community-based settings, such as schools. To improve the nutrition behaviours of primary-school aged children, consistent with dietary guideline recommendations, three LHDs sought to engage with schools, within their respective regions, to adopt an effective school-based nutrition intervention to support parents to improve the foods packed within children’s lunchboxes. ‘SWAP IT’ is a healthy lunchbox program developed by LHD health promotion staff in collaboration with researchers and end-users (e.g. parents, teachers, principals). It consists of brief messages delivered to parents via existing school-parent communication channels (e.g. app, newsletter or social media) to encourage parents to ‘swap’ energy-dense nutrient-poor ‘discretionary’ food items packed within children’s lunchboxes to nutrient-dense food items (i.e. ‘everyday’ or core foods). Previous randomised trials of SWAP IT have shown the program to be effective in improving child nutrition and weight outcomes, as well as being highly acceptable to parents and principals and at low-cost [18–20].

To address the limitations of the existing evidence-base, we sought to conduct collaborative research embedded within Health Promotion Units of NSW LHDs to support the adoption of SWAP IT in schools and accelerate the generation and use of implementation research for improvement. Specifically, the three LHD Health Promotion teams collaborated to facilitate the large-scale adoption of SWAP IT within their jurisdictions. The LHDs differ in terms of the characteristics of the community they serve, their geographical and population size, and available resources. To collectively inform future efforts to implement and maximise adoption of the SWAP IT program, within each LHD and across other NSW LHDs, a collaboration was formed where the evaluations and learnings from each LHD would be shared [21]. Specifically, structures were established to facilitate collaboration and an exchange of evidence amongst LHDs whilst still allowing for tailoring to suit local context and health promotion team infrastructure [21]. This was operationalised consistent with a Learning Health System perspective of improvement and included a Community of Practice (CoP). The CoP provided a structure for i.) shared governance; ii.) the harmonisation of key research methods, enabling direct comparison between evaluations via a collaborative network trial, and iii.) supporting collective learning, knowledge exchange and health service improvement [21]. As such, it served to support the generation and sharing of new knowledge to advance practice, and was underpinned by a commitment of members to work collectively and constructively to do so [21].

In this context, the primary aim of this paper is to describe and report the outcomes of implementation strategies employed by each LHD to improve the adoption of SWAP IT. We also discuss and reflect on learnings from an innovative approach to enhance implementation research production for the advancement of public health nutrition via the use of a collaborative network of RCTs.

## Methods

### Context

Health promotion practitioners from the three LHDs and researchers from the National Centre of Implementation Science (NCOIS), a collaborating research centre of which the three LHDs are research and translation partners, established a CoP with the shared purpose of maximising school adoption of SWAP IT. The development and function of the CoP has been described elsewhere [21]. Health promotion staff within each LHD were responsible for employing strategies, tailored to align with their service plans, resources and staffing, to maximise adoption of SWAP IT within their respective regions. LHD health promotion staff were also responsible for participating in and engaging with activities of the CoP; and contributing to the sharing and exchange of learnings to improve approaches to maximise school adoption of the SWAP IT program. NCOIS provided funding for staffing, infrastructure and co-ordination support for the CoP. Researchers from NCOIS facilitated workshops, implementation check-ins, knowledge exchange meetings and provided additional support to LHDs on request. NCOIS was also responsible for leading the evaluation of the CoP in partnership with the participating LHDs, including: selecting the study design and trial outcomes; obtaining ethics approval; confirming the study sample and conducting the randomisation with an independent statistician; overseeing the delivery of the scale-up strategies; and conducting the data collection component.

### Study design and setting

The novel randomised trial design, which draws on principles of Master Protocols and was referred to as a “Collaborative Network Trial” by the research team, was employed to evaluate the effectiveness of strategies employed by each LHD to maximise the adoption of SWAP IT. Master Protocols, traditionally used to test pharmacological interventions, refer to trial designs employing co-ordinated approaches and centralised trial infrastructure to assess the effects of interventions [22–24]. This infrastructure typically includes a centralised trial protocol and governance, with standardised study procedures for recruitment, evaluation, data collection and analysis, and reporting [22–24].

Within this Collaborative Network Trial, three independent, two arm parallel group randomised controlled trials (RCT) (one per LHD) were conducted simultaneously to examine the potential effectiveness of various combinations of strategies on school adoption of SWAP IT. The key trial methods, measures and data collection processes were harmonised providing individual school-level data for planned pooled analyses. The trials were prospectively registered with Australia New Zealand Clinical Trials Register (ACTRN12622000257763; ACTRN12622000406707; ACTRN12622000252718) and align with the CONSORT reporting guidelines for randomised controlled trials (Supplementary File 1). Ethics approval was obtained from the Hunter New England Human Research Ethics Committee (Ref. No. 2019/ETH12353), the University of Newcastle (Ref. No. H-2008–0343), the NSW State Education Research Application Process (Ref. No. 2018247) and the Maitland-Newcastle Catholic Schools Office.

Primary schools, which typically enrol children aged 5–12 years, from the Independent, Department of Education and Catholic school sectors, located within the three NSW LHDs served as the sampling frame. The three LHDs have approximately 600 primary schools across these sectors, and encompass socioeconomically and geographically diverse regions [25].

### Study population and recruitment

A list of potentially eligible schools located within the three LHDs was obtained by health promotion staff within each LHD and provided to NCOIS to confirm eligibility of the study sample. Schools were considered eligible if they: were a primary or combined school located within the three participating LHDs that catered for at least one primary school year; had not previously implemented SWAP IT; and had not participated in any previous trials evaluating the effectiveness of SWAP IT.

Schools with secondary students only, schools for specific purposes (e.g. schools catering exclusively for children requiring specialist care) and schools who had already implemented SWAP IT were ineligible to participate. All schools that met the eligibility criteria were included in the study.

### Randomisation and blinding

Prior to the delivery of the strategies, schools within each LHD (hereafter named LHD 1,2 or 3) were randomly allocated, following a block randomisation procedure (block sizes 2–6) in a 1:1 ratio, to either High Intensity (HI) or Business as Usual (BAU), using a computerised random number function in Microsoft Excel 2013. Randomisation was conducted by an independent statistician and overseen by NCOIS. Randomisation was stratified by school geographic and socioeconomic status (SES), given its association with the implementation of school nutrition programs [26], as determined by the Socio-Economic Indexes for Areas categorisation using school postcodes.

School staff were not aware of group allocation. However, LHD health promotion staff delivering the strategies and research staff conducting the evaluation were not blinded to group allocation. The health promotion units of all three LHDs had been working in school-based health promotion for over a decade and staff were experienced in engaging with schools. Similarly, all health promotion units had a shared interest and commitment from the unit Director to support adoption of SWAP IT by schools in their region. Each LHD served populations inclusive of metropolitan centres, and rural and remote communities. However, the health promotion unit of LHD 1, served a population three to four times larger than those in LHDs 2 and 3; and had significantly greater capacity to undertaken school-based health promotion, with a workforce more than five times larger. Each health promotion team had experience in engaging in research.

### Implementation strategies

To inform the development of strategies employed by LHDs to encourage school adoption of SWAP IT, each of the three LHDs participated in a series of group workshops facilitated by public health researchers from NCOIS. The workshops followed a structured process based on the NSW Scalability Guide [27] to enable LHD health promotion staff to develop and/or select a series of strategies to implement within their region. Each strategy was tailored to the context of their region, usual service delivery approach and capacity of the health promotion workforce. While usual service delivery (implementation support) approaches varies between health promotion units, and depending on the interventions to be implemented and the settings where this is occurring, they would commonly use strategies such as the use of educational meetings including training, provision of educational materials, outreach visits and facilitation, consensus processes, and audit and feedback [28, 29]. Strategies were developed to target barriers to the adoption of SWAP IT and other school-based nutrition interventions, identified through previous evaluations of the program and literature searching. The key barriers identified included school and principal awareness of the program, perceived workload for school staff to implement the program, and the role/responsibility of schools in promoting healthy lunchboxes.

**Fig. 1:**
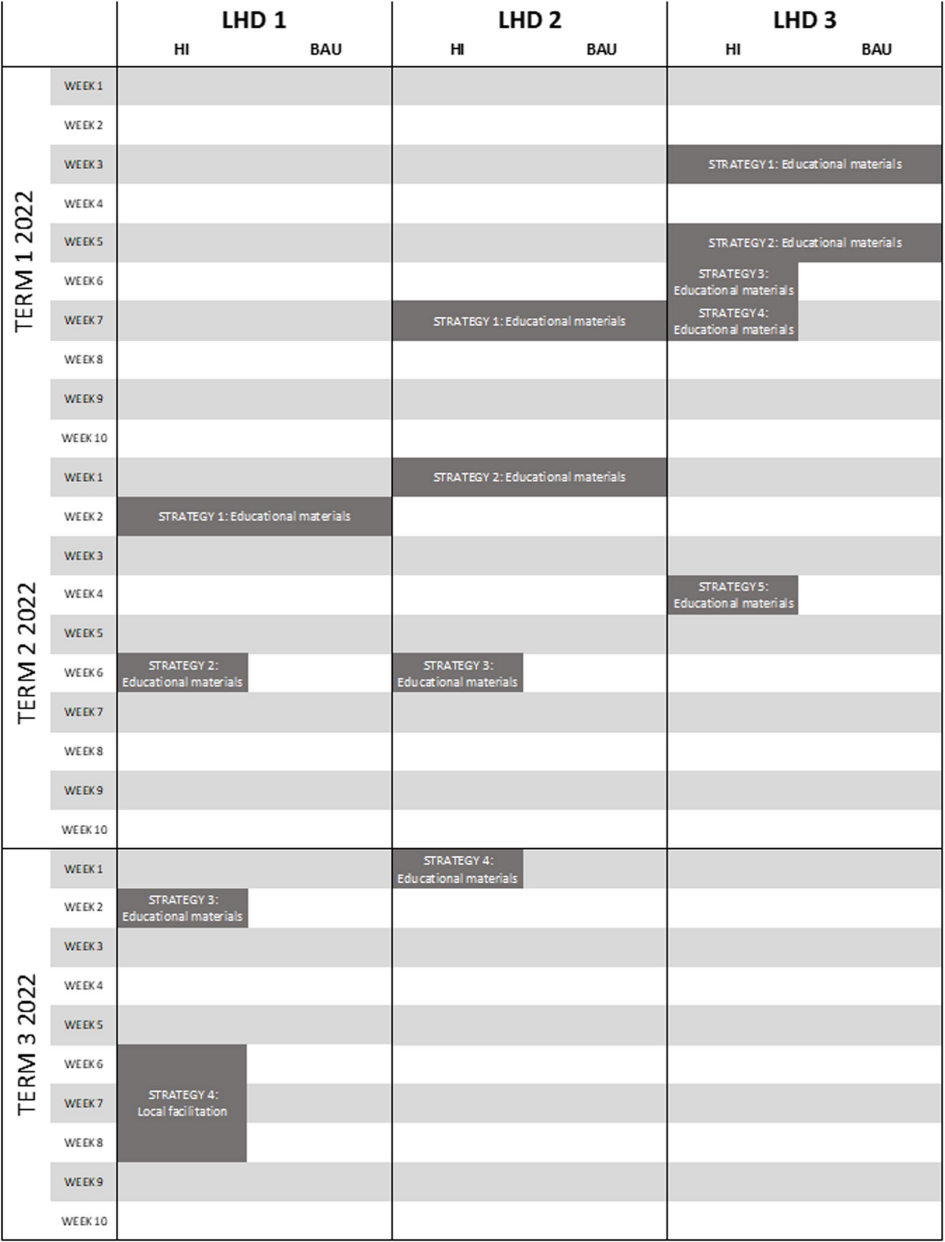
Timeline of delivery of implementation strategies across each LHD

Strategies were categorised using the Expert Recommendations for Implementing Change (ERIC) taxonomy [30] and are summarised in Table 1 using the Proctor framework [31] to enable replication. The timeline for delivery of each strategy across the three LHDs is provided in Fig. 1. All strategies employed by LHDs consisted of a link to the SWAP IT website where schools were able to register for the program. To register for SWAP IT, schools completed a two-minute online registration form to confirm their school details and select how they plan to implement SWAP IT (e.g. via a communication app, newsletter or social media). Following registration (i.e. adoption of SWAP IT), schools did not receive any further implementation strategies promoting the adoption of SWAP IT, but instead received support by the LHD to implement SWAP IT and other health promotion programs as part of usual service delivery.

**Table 1:** Implementation strategies employed by each LHD

Strategy	LHD 1	LHD 2	LHD 3
Strategy 1	**Educational materials** ***Actor:*** LHD health promotion staff ***Action:*** Email to central school email address, and direct Principal or Key Contact email address where known, specifically promoting SWAP IT as an exciting new program to improve student lunchboxes. A video (video one) providing an overview of SWAP IT and the time required to implement the program was embedded within the email ***Target:*** Principals and Key School Contacts in HI and BAU groups ***Temporality:*** Term 2, Week 2 (4/5/2022) ***Dose:*** Once during the study period	**Educational materials** ***Actor:*** LHD health promotion staff ***Action:*** A 14-page Health Promoting Schools newsletter emailed to the central school email address to promote LHD health promotion programs. The newsletter is distributed each term to primary and secondary schools and showcases the range of health promotion programs available within the LHD. SWAP IT information was presented across two-thirds of page four and contained a link to video one (described in LHD 1 Strategy 1) ***Target:*** Principals and Key School Contacts in HI and BAU groups ***Temporality:*** Term 1, Week 7 (11/3/2022) ***Dose:*** Once during the study period	**Educational materials** ***Actor:*** LHD health promotion staff ***Action:*** A two-page calendar promoting LHD health promotion programs and a link to an online survey emailed to the central school email address. The calendar is an annual resource that contains links to available health programs within the LHD, including SWAP IT. The survey enables schools to provide up-to-date contact details to the LHD and links schools to available programs, including SWAP IT ***Target:*** Principal and Key School Contacts in HI and BAU groups ***Temporality:*** Term 1, Week 3 (8/2/2022) ***Dose:*** Once during the study period
Strategy 2	**Educational materials** ***Actor:*** LHD health promotion staff ***Action:*** Email to central school email address, and direct Principal or Key Contact email address where known, specifically promoting SWAP IT as “free, easy to run and delivers healthier lunchboxes for kids”. A video (video two) describing the ease of the program to implement and expected workload on staff was embedded within the email ***Target:*** Principals and Key School Contacts in HI group only ***Temporality:*** Term 2, Week 6 (1/6/2022) ***Dose:*** Once during the study period	**Educational materials** ***Actor:*** LHD health promotion staff ***Action:*** Email to central school email address specifically promoting SWAP IT and offering free resources for supporting parents to pack healthy lunchboxes for schools. These free resources included parent booklets and classroom resources. Video one (described in LHD 1 Strategy 1) was embedded in the email ***Target:*** Principals and other teaching staff in HI and BAU groups ***Temporality:*** Term 2, Week 1 (26/4/2022) ***Dose:*** Once during the study period	**Educational materials** ***Actor:*** LHD health promotion staff ***Action:*** An e-newsletter emailed to the central school email address to promote LHD health promotion programs. The e-newsletter promoted six programs available to schools within the LHD. SWAP IT appeared second ***Target:*** Principals and Key School Contacts in HI and BAU groups ***Temporality:*** Term 1, Week 5 (23/2/2022) ***Dose:*** Once during the study period
Strategy 3	**Educational materials** ***Actor:*** LHD health promotion staff ***Action:*** Email to central school email address, and direct Principal or Key Contact email address where known, specifically promoting SWAP IT and offering free resources for kinder orientation for schools. Free resources included a SWAP IT parent booklet and a Crunch&Sip brochure. A video (video three) describing why healthy lunchboxes are important for child health and wellbeing was embedded within the email ***Target:*** Principals and Key School Contacts in HI group only ***Temporality:*** Term 3, Week 2 (27/7/2022) ***Dose:*** Once during the study period	**Educational materials** ***Actor:*** LHD health promotion staff ***Action:*** Email to central school email address specifically promoting SWAP IT as being “free, easy to run and delivers healthier lunchboxes for kids”. Videos one and two (described in LHD 1) were embedded in the email ***Target:*** Principals and other staff in HI group only ***Temporality:*** Term 2, Week 6 (31/5/2022) ***Dose:*** Once during the study period	**Educational materials** ***Actor:*** LHD health promotion staff ***Action:*** Email to central school email address specifically promoting SWAP IT and offering free electronic resources to support parents to pack healthy lunchboxes. Video one (described in LHD 1) was embedded in the email ***Target:*** Principals in HI group only ***Temporality:*** Term 1, Week 6 (1/3/2022) ***Dose:*** Once during the study period
Strategy 4	**Local facilitation** ***Actor:*** LHD health promotion staff ***Action:*** Phone call to central school phone number, requesting to speak with the Principal or Key Contact. Aim of the phone call was to introduce SWAP IT to schools (if they weren’t familiar with the program) and address school identified barriers to adoption ***Target:*** Principals and Key School Contacts in HI group ***Temporality:*** Term 3, Weeks 6 – 8 (23/8/2022 – 9/9/2022) ***Dose:*** Once during the study period	**Educational materials** ***Actor:*** LHD health promotion staff ***Action:*** Email to central school email address specifically promoting SWAP IT by highlighting why lunchboxes matter and offering free kindy orientation resources for schools. Videos one and two (described in LHD 1) were embedded in the email ***Target:*** Principals and other staff in HI group only ***Temporality:*** Term 3, Week 1 (20/7/2022) ***Dose:*** Once during the study period	**Educational materials** ***Actor:*** LHD health promotion staff ***Action:*** Email to central school email address specifically promoting SWAP IT as “free, easy to run and delivers healthier lunchboxes for kids”. Videos one and two (described in LHD 1) were embedded in the email ***Target:*** Principals in the HI group only ***Temporality:*** Term 1, Week 7 (8/3/2022) ***Dose:*** Once during the study period
Strategy 5			**Educational materials** ***Actor:*** LHD health promotion staff ***Action:*** Email to central school email address specifically promoting SWAP IT by highlighting the link between healthy lunchboxes and learning. All three videos (described in LHD 1) were embedded in the email ***Target:*** Principals in the HI group only ***Temporality:*** Term 2, Week 4 (17/5/2022) ***Dose:*** Once during the study period

### Control

The implementation strategies delivered to schools allocated to the BAU group across LHDs is described in Table 1. For all BAU groups, this consisted of a singular strategy delivered one to two times. To minimise contamination, execution of the implementation strategies was centrally monitored by the research team in consultation with health promotion staff from each LHD.

## Outcomes and data collection methods

To aid the comparison of findings across LHDs, the study outcomes and measures were harmonised across the three RCTs. The measures used to assess each study outcome are described below:

### Primary outcome

Adoption of the SWAP IT program was defined as the proportion of schools in each trial arm who agreed to implement SWAP IT. This was assessed via registrations automatically captured through the SWAP IT website once a school registers for the program (i.e. completes the online registration form) at 6-months following the delivery of the first strategy for each LHD.

### Secondary outcomes

Acceptability of the strategies, defined by the perception among implementation stakeholders (school staff), that a given strategy is agreeable, palatable or satisfactory, and engagement with the strategies [32], was assessed via an online or telephone survey conducted with school principals 6-months following the delivery of the first strategy for each LHD. A random subsample of schools from LHD 1 (due to the larger number of schools in LHD 1), in addition to all schools in LHD 2 and 3 were invited to participate in the survey (182 schools in total). Principals were asked if they recalled receiving each of the strategies (e.g. telephone call and/or email from health promotion staff) during the study period. For strategies the participants recalled receiving, they were asked to rate how acceptable they found the strategy on a 5-point Likert scale (1 = highly unacceptable; 5 = highly acceptable). Participants were also asked to rate how acceptable other potential strategies (that were not utilised in the current study) would be if they were delivered to schools on a 5-point Likert scale (1 = highly unacceptable; 5 = highly acceptable).

Barriers and facilitators to school adoption of SWAP IT were assessed via an online or telephone survey conducted with school principals as described above. Participants were asked to select, from a pre-specified list, what was their main barrier and enabler to adopting the SWAP IT program. Participants were also given the option to add their own barrier and enabler if they selected ‘other’.

School characteristics, including school sector, geolocation, number of student enrolments, proportion of Aboriginal or Torres Strait Islander students and proportion of students with a language background other than English were sourced from publicly available information via the Australian Curriculum, Assessment and Reporting Authority (ACARA) database [33].

## Sample size and data analysis

Descriptive statistics were used to describe school characteristics, adoption of the nutrition program, intervention fidelity, acceptability and engagement with the adoption strategies and barriers and enablers to school adoption of the program.

Analysis of trial outcomes were undertaken under an intention to treat framework, whereby data from schools is analysed in the group for which it was randomly assigned. Firstly, for assessment of school-level program adoption for each trial (i.e. the primary trial outcome), between group differences were assessed using logistic regression analyses. The model included a term for treatment group (HI vs BAU). There were no missing data at follow-up for the primary outcome of adoption. All statistical tests were 2 tailed with alpha of 0.05.

Assuming a 10% adoption rate of the program in the comparison group (based on prior pilot data of the research team) [34], a sample size of approximately 30 schools per group would be sufficient to detect an absolute difference between groups of 30%, with 80% power and an alpha of 0.05. An effect size of 30% is consistent with that achieved for the implementation of other school-based nutrition programs [35]; and within the range of improvements reported in implementation of health promotion programs in schools more broadly [36]. The targeted effect size was also considered to be of benefit from a population level perspective, exposing tens of thousands of students to the effective SWAP IT program and improving the nutritional quality of hundreds of thousands of packed student lunches each week, if implemented across participating jurisdictions. Further, it was considered acceptable to participating units.

Secondly, we conducted multiple meta-analyses to pool the effects of different trial arms across the three trials, allowing us to compare and rank the potential relative effects (via direct or indirect comparisons) of the different strategies employed by each LHD on the primary trial outcome (i.e. SWAP IT adoption). This included a comparison of: i.) the effects of educational materials alone (direct effect) compared to BAU; ii.) the effect of educational materials and local facilitation (direct effect) compared to BAU; and iii.) the relative effect of educational materials and local facilitation compared to educational materials alone (indirect effect). For each meta-analysis, we pooled data using generic inverse variance method using Review Manager 5 software (RevMan) using a random-effect model. Odds ratios and confidence intervals were calculated for each analysis.

## Results

### Sample characteristics

**Fig. 2:**
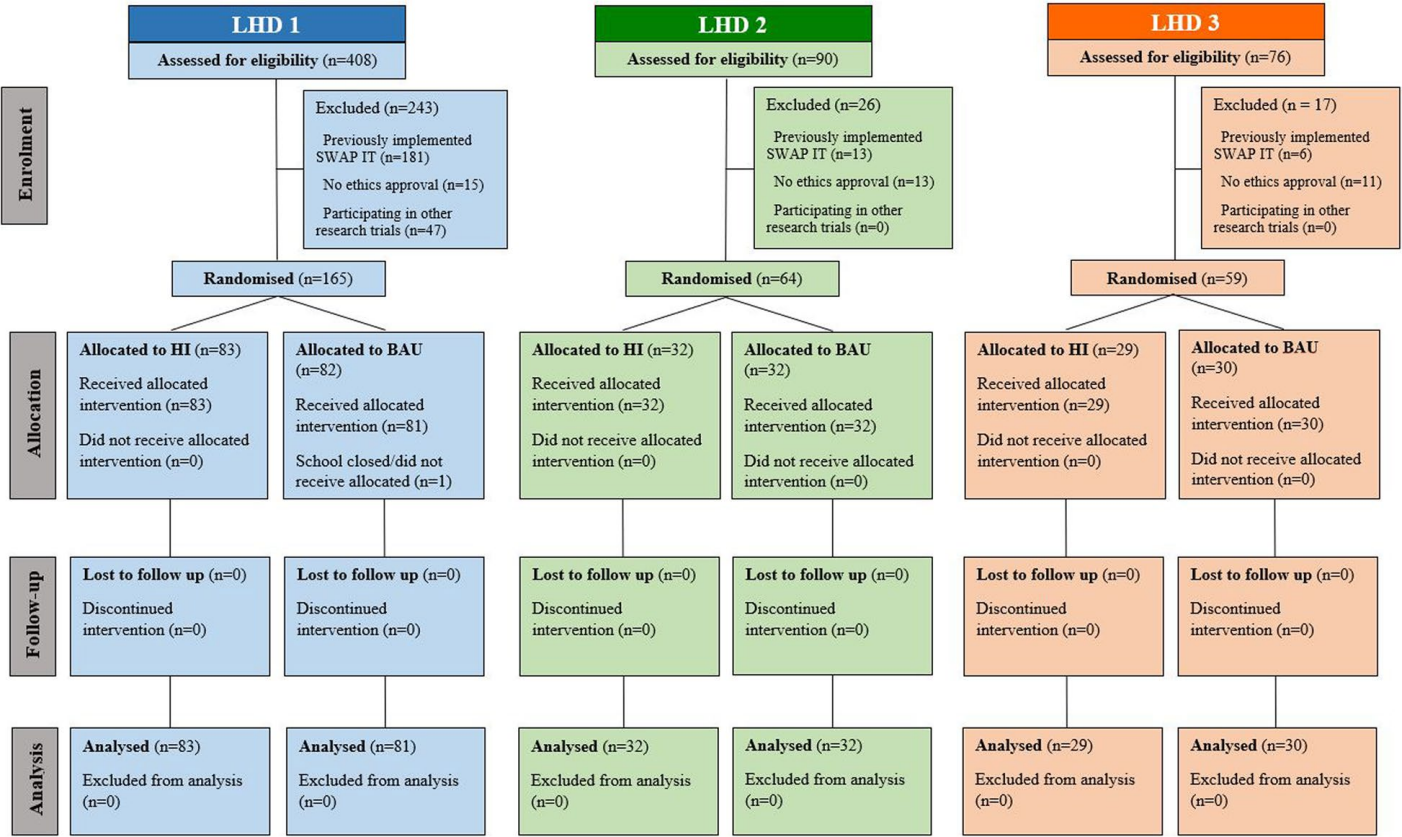
Study flow diagram

A total of 287 schools across the three LHDs were included in the study: LHD 1, *n* = 164 (HI *n* = 83; BAU *n* = 81); LHD 2, *n* = 64 (HI *n* = 32; BAU *n* = 32); and LHD 3, *n* = 59 (HI *n* = 29; BAU *n* = 30) (Fig. 2). Overall, the majority of schools were from the Government sector (*n* = 220, 76.7%), and were located in major cities (*n* = 100, 35.1%) and inner regional areas (*n* = 102, 35.5%). The mean number of enrolled students was 282.3 (SD 280.1). Characteristics of the included schools, overall and per LHD, are summarised in Table 2.

**Table 2:** Sample characteristics

	LHD 1			LHD 2			LHD 3			Overall
	HI^b^	BAU^c^	Total	HI	BAU	Total	HI	BAU	Total	Total
**Total, n**	83	81	164	32	32	64	29	30	59	287
**Sector, n (%)**										
Government	64 (77.1)	53 (65.4)	117 (71.3)	27 (84.4)	27 (84.4)	54 (84.4)	24 (82.8)	25 (83.3)	49 (83.1)	220 (76.7)
Non-Government	19 (22.9)	28 (34.6)	47 (28.7)	5 (15.6)	5 (15.6)	10 (15.6)	5 (17.2)	5 (16.7)	10 (16.9)	67 (23.3)
**Geolocation, n (%)**^**a**^										
Major Cities	24 (28.9)	24 (29.6)	48 (29.3)	0	0	0	26 (89.7)	26 (86.7)	52 (88.1)	100 (35.1)
Inner regional	30 (36.1)	30 (37.0)	60 (36.6)	19 (59.4)	17 (53.1)	36 (56.3)	2 (6.9)	4 (13.3)	6 (10.2)	102 (35.5)
Outer regional	25 (30.1)	25 (30.9	50 (30.5)	12 (37.5)	15 (46.9)	27 (42.2)	0	0	0	77 (27.0)
Remote/very remote	2 (2.4)	2 (2.5)	4 (2.4)	1 (3.1)	0	0	0	0	0	5 (1.7)
**Student enrolments, mean (SD)**^**a**^	227.1 (235.6)	264.7 (285.5)	245.9 (261.6)	207.7 (172.8)	218.8 (268.4)	213.2 (224.0)	455.4 (311.9)	464.3 (329.1)	460.0 (318.3)	282.3 (280.1)
**Proportion of Aboriginal and/or Torres Strait Islander students, average % (range)**^**a**^	18.5 (0–67)	21.0 (0–100)	19.7 (0–100)	21.5 (0–78)	15.8 (0–100)	18.2 (0–100)	9.9 (0–24)	12.3 (1–100)	11.1 (0–100)	17.7 (0–100)
**Proportion of students with language background other than English, average % (range)**^**a**^	6.5 (0–31)	7.8 (0–38)	8.5 (0–38)	10.0 (0–49)	5.6 (0–17)	7.8 (0–49)	11.3 (0–23)	11.3 (0–23)	11.3 (0–23)	8.1 (0–49)

^a^ Data missing for 3 schools (2 schools in LHD 1 and 1 school in LHD 3)

^b^HI = high intensity

^c^BAU = business as usual

### Adoption

**Fig. 3:**
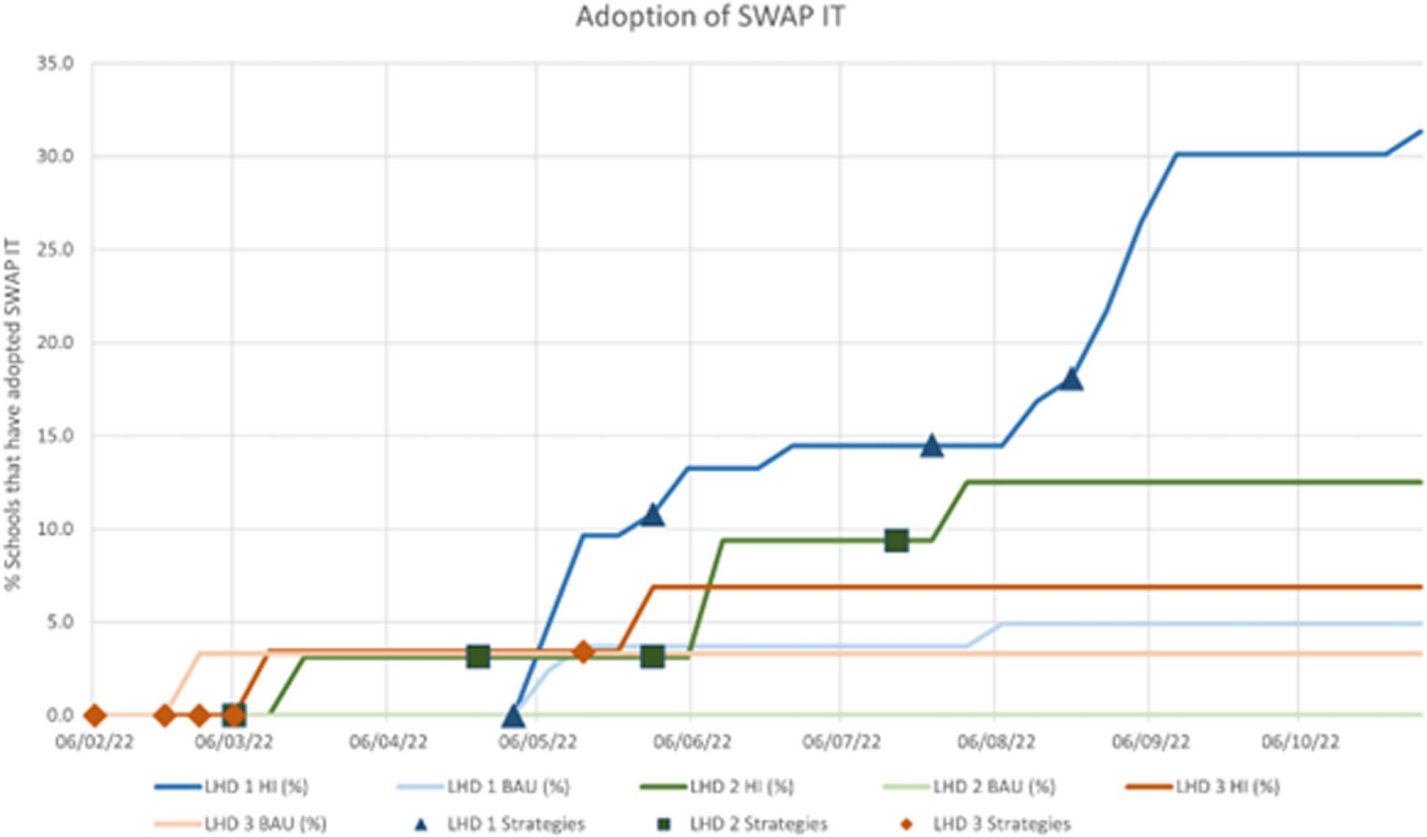
Adoption of SWAP IT across the three LHDs following the delivery of each strategy

For LHD 1, schools allocated to the HI group had 8.78 times the odds of adopting SWAP IT (OR 8.78; 95%CI 2.90, 26.56; p < 0.001) relative to schools that were allocated to the BAU group. There were no significant differences in adoption between groups for LHD 2 (OR 5.74; 95%CI 0.94, ∞; *p* = 0.11) or LHD 3 (OR 2.15; 95%CI 0.18, 25.07; *p* = 0.54) (Fig. 3).

Findings of the meta-analyses indicated the combined accumulative effect of the strategies employed across the three studies resulted in moderate improvements in school adoption of SWAP IT (OR 7.21; 95%CI 2.77, 18.75; *n* = 3 studies). When comparing the relative effective of the different strategies on adoption, the meta-analyses indicated that the combination of educational materials and local facilitation strategies compared to BAU resulted in moderate improvements in adoption (OR 8.78; 95%CI 2.90, 26.56; *n* = 1 study; direct effect). Educational materials compared to BAU (OR 4.06; 95%CI 0.61, 26.93; *n* = 2 studies; direct effect); and the combination of local facilitation and educational materials compared to educational materials alone (OR 4.18; 95%CI 1.60, 10.04; *n* = 3 studies; indirect effect) both resulted in smaller improvements in adoption.

### Acceptability of the implementation strategies

Of the 73 schools across the three LHDs that completed or partially completed the survey (40.1% of invited participants), 18 participants recalled receiving an email regarding SWAP IT within the last 6 months (24.7% of survey respondents), 14 of which (77.8%) reported this strategy as acceptable. Of the four participants that recalled receiving a telephone call regarding SWAP IT within the last 6-months (5.5% of survey respondents), no schools reported this strategy as acceptable.

Survey participants indicated that receiving information about registering for SWAP IT through the following channels would be acceptable: via email directly to school Principal (*n* = 33, 51.6% of survey respondents), via email to school office (*n* = 57, 90.5% of survey respondents), telephone call to school office (*n* = 32, 50.0% of survey respondents), online webinars, training and information sessions (*n* = 45, 70.3% of survey respondents) and face-to-face meetings, workshops or conferences (*n* = 29, 45.3% of survey respondents). Additionally, receiving information from the following sources was considered acceptable: information and materials from the Local Health District (local health promotion staff) (*n* = 55, 85.9% of survey respondents); information and materials from professional organisations (*n* = 41, 64.1% of survey respondents); information and materials from education stakeholders such as school directors (*n* = 39, 60.9% of survey respondents); and endorsement from peers and opinion leaders (*n* = 35, 54.7% of survey respondents).

### Barriers and enablers to school adoption of SWAP IT

The most frequently reported barrier to school adoption of SWAP IT was expected workload for staff (*n* = 25, 37.3% of survey respondents), followed by perception that parents and carers don’t think it is the school’s place to provide nutrition information (*n* = 11, 16.4% of survey respondents) and food insecurity is a greater priority for my community (*n* = 5, 7.5% of survey respondents). Ten schools (14.9% of survey respondents) reported that there were no barriers to adopting SWAP IT. The most commonly reported enablers to school adoption of SWAP IT were keep the program free (*n* = 22, 32.8% of survey respondents), support for the program from teachers (*n* = 6, 9.0% of survey respondents), alignment with school plan and curriculum (*n* = 6, 9.0% of survey respondents) and show evidence that SWAP IT supports development of healthy habits in children (*n* = 5, 7.5% of survey respondents). A summary of the most commonly reported barriers and enablers to school adoption of SWAP IT by LHD is provided in Table 3.

**Table 3:** School barriers and enablers to adopting SWAP IT

	LHD1^a^n	LHD 2^b^n	LHD 3^**c**^n	Overall ^d^n
**Barriers**				
Expected workload for staff	6 (28.6)	13 (48.1)	6 (31.6)	25 (37.3)
Perception that parents and carers don’t think it’s the schools place to provide nutrition information	2 (9.5)	2 (7.4)	7 (36.8)	11 (16.4)
There are no barriers	5 (23.8)	3 (11.1)	2 (10.5)	10 (14.9)
Food insecurity is a greater priority for my community	2 (9.5)	1 (3.7)	2 (10.5)	5 (7.5)
Concern about delivery mode	2 (9.5)	1 (3.7)	1 (5.3)	4 (6.0)
**Enablers**				
Keep the program free	7 (33.3)	7 (25.9)	8 (42.1)	22 (32.8)
Support for the program from teachers	1 (4.8)	4 (14.8)	1 (5.3)	6 (9.0)
Alignment with the school plan and curriculum	2 (9.5)	4 (14.8)	0 (0)	6 (9.0)
Evidence that SWAP IT supports the development of healthy habits in children	2 (9.5)	1 (3.7)	2 (10.5)	5 (7.5)
Support for the program from parents	2 (9.5)	1 (3.7)	2 (10.5)	5 (7.5)
Alignment with health and wellbeing priorities	2 (9.5)	2 (7.4)	0 (0)	4 (6.0)
Make the registration process easy	1 (4.8)	3 (7.4)	0 (0)	4 (6.0)

^a^*n* = 21 schools from LHD 1 completed this item

^b^*n* = 27 schools from LHD 2 completed this item

^c^*n* = 19 schools from LHD 3 completed this item

^d^*n* = 67 schools overall completed this item

## Discussion

This paper describes a study employing a novel collaborative network trial design, consisting of three independent randomised controlled trials with a harmonised evaluation, to evaluate the effectiveness of strategies to improve school adoption of SWAP IT across three NSW LHDs. Educational materials were employed as a strategy across all three LHDs, whilst local facilitation was employed by one LHD to maximise adoption of the program. Findings of the meta-analysis indicated that the combination of educational materials and local facilitation was the most potent approach to increasing school adoption of SWAP IT across the three LHDs (OR 8.78; 95%CI 2.90, 26.56).

Whilst the effect size uncertain (OR 4.06; 95%CI 0.61, 26.93; *n* = 2 studies), findings of the meta-analyses suggest that delivering educational materials as an isolated strategy (i.e. not in combination with other strategies) may represent an efficient, acceptable and inexpensive means of making small improvements in school adoption of SWAP IT [9, 37, 38]. However, in order to achieve substantial absolute changes in adoption, additional more comprehensive strategies may be required. This is supported by previous systematic reviews findings that have found larger effects when educational materials are employed as part of more comprehensive implementation strategies [9, 37, 38]. Methods of ensuring principals were exposed to the education material may also enhance its effects [39]. For example, in the absence of direct email addresses of some school principals, education materials were often emailed to school administration staff relying on them to forward the material to the principal. As such, many principals may not have received the educational materials as intended. The distribution of materials direct to principals, or from a more salient source, such as the Department of Education, may have improved principal receipt and engagement with education materials. Amendment of the materials to emphasise factors identified in this study as facilitators to program adoption, such as the program being free, and its alignment with the curriculum may also enhance its acceptability and impact. Finally, the delivery of education materials in this study occurred during a period of disruption to usual engagement with schools due to COVID-19 restrictions. This is a unique contextual factor that no doubt limited the opportunity for education materials to reach school decision makers or their capacity to act on them.

Promisingly, the local facilitation strategy, employed by LHD 1, resulted in a substantial increase in adoption of SWAP IT amongst schools that received the strategy. This strategy directly targeted the principal or other decision maker via a tailored telephone call conducted by health promotion staff to discuss school barriers to adopting the program. The effectiveness of this local facilitation strategy is consistent with a pilot of this strategy within a previous evaluation of SWAP IT, which resulted in an increase in adoption of 24% [34], in addition to the broader literature which has found that local facilitation can lead to end-users being almost three times as likely to adopt innovations [40]. This is likely to be in part due to local facilitation allowing for more tailored approaches to health promotion; enabling staff to discuss and address school-specific barriers to adopting programs with school decision makers [40].

While effective, school staff that recalled receiving a local facilitation call indicated it was not an acceptable strategy. The findings may suggest the strategy is inherently unacceptable, perhaps because of the time required for school staff to engaged in phone calls. However, it may also suggest the strategy was not executed in a way that was perceived as beneficial for schools. Prior research suggests a range of personal characteristics, interpersonal skills and confidence are important for local facilitation [41], as are behaviours such as, active listening, clear communication of goals, clear directions for activity, keeping activity on-task, affirmation, and professional respect [42]. In this study, we did not assess the extent to which such attributes, or behaviours were present among school facilitators. Nonetheless, consideration of these factors in facilitator recruitment, training and support may improve the acceptability and impact of this strategy [41]. Further research is warranted to investigate this hypothesis.

Given the substantial variation in workforce capacity and available resources across NSW LHDs, the ability of health promotion staff to deliver high intensity strategies (i.e. local facilitation) to all schools may be limited. Indeed, unlike the health promotion unit in LHD 1, staff availability and competing priorities were among the reasons local facilitation was not included as a strategy to support adoption of SWAP IT among units in LHD’s 2 and 3. Local facilitation via in-person or telephone contact with schools, however, is a common strategy employed by many NSW health promotion units to support schools to adopt healthy eating and physical activity promoting policies and practices more broadly [43]. The findings provide strong evidence supporting its use to support the adoption of SWAP IT also. However, other strategies less reliant on staff capacity with the capacity to address a range of school barriers to program adoption could also be considered. For example, the use of digital decision support tools in education settings have led to improvements in implementation of nutrition programs at minimal cost [44].

Findings from the survey conducted with Principals provide useful insights into the strategies that could potentially be employed to maximise adoption of SWAP IT and other school-based health promotion programs more broadly. Several additional strategies (not included in the current study) were also considered to be highly acceptable to Principals, including webinars, training and information sessions, and information and materials provided by educational stakeholders and health professionals. Interestingly, schools considered an email to the central school email address (i.e. educational materials) to be highly acceptable, whilst a telephone call from health promotion staff (i.e. local facilitation) was not, suggesting that there may be contradiction between the strategies that are acceptable to schools and those that are effective in increasing adoption of school-based health promotion programs. Co-designing strategies to maximise adoption of effective school-based health promotion programs with end-users, such as Principals and other school staff, in addition to researchers and health promotion practitioners, should be considered prior to efforts to scale programs in order to ensure strategies are both acceptable and effective in maximising school adoption of such programs [45].

The novel use of a collaborative network trial design to test the effectiveness of strategies employed by different LHDs to maximise school adoption of SWAP IT, provided ameans to simultaneously test the effect of different combinations of strategies. The design draws on a number on principles of master protocol designs. It also adopts methods including measure harmonisation, and independent trial collaborations, and analytic techniques employed in established designs such as prospective meta-analyses [46]. Such an approach has the potential to address several limitations of more commonly used approaches to knowledge and research production in school-based health promotion research. First, the trial provided critical research infrastructure for improvement via NCOIS that was not otherwise available to health promotion units. This presented an opportunity for health promotion units within each LHD to draw on this for local evaluation of their implementation strategies. As such the trial allowed data regarding their effects of local strategies be captured that would otherwise not have been subject to rigorous evaluation.

Second, the design allowed health promotion staff within each LHD to select and tailor their strategies to align with local context and infrastructure. This flexibility was a key factor in health promotion team engagement in the trial, and yielded natural variability in the strategies employed, and tested across each LHD. Further the the harmonised data collection and outcome methods provided an opportunity to make inferences about the effects of different strategies and contexts. While more elegant and statistically efficient research designs, such as multi-site RCTs or factorial RCTs, would provide a more rigorous assessment of the comparative effects of different implementation strategies [47], their requirements for standardisation of strategies being tested (across LHDs) was not acceptable to health promotion units. The collaborative network trial design represented a pragmatic alternative allowing the execution of implementation strategies to be managed locally by health promotion staff and integrated into their usual project management processes.

Finally, a feature of the research that was particularly valued by health promotion teams was the opportunity for knowledge exchange and learning from other health promotion units. The primary vehicle for this was the CoP. We have published health promotion unit reflections on the CoP elsewhere [21], demonstrating that it was a valued means of supporting research, health promotion collaboration, sharing and practice improvement.

A number of limitations of the research warrant consideration. The network included just three RCTs, limiting our capacity to formally quantify and contrast the effects of different implementation strategies employed across different LHDs or examine effect modifiers. While indirect comparisons of strategy effects between LHDs was considered useful, such comparisons are likely confounded. In a second phase of this project, however, the network has expanded to now include RCTs undertaken in ten LHDs, encompassing approximately 90% of NSW. This enables formal quantitative analytical methods such as individual patient data network meta-analyses [48] to assess the effects of a broader variety of strategies, and strategy combinations across different contexts. It may also enable some identification of, and potential control for measured confounding variables. It also speaks to the potential feasibility of this approach to generating evidence to support system-wide improvements in the adoption and implementation of health initiatives. A further limitation of the study is the absence of measurement of determinants of SWAP IT implementation. Strategies employed by health promotion units were informed by the Theoretical Domains Framework (TDF) [49]. Measurement of TDF domains would have enabled a better understanding of the mechanisms of action and of any differences in effects between LHDs.

## Conclusions

This paper outlines a collaborative network trial conducted across three NSW LHDs to evaluate strategies for enhancing school adoption of the SWAP IT program. Combining educational materials and local facilitation proved most effective, significantly increasing adoption rates. While educational materials alone showed some improvement, more comprehensive strategies may be necessary for substantial change. This novel trial design allowed for the tailoring of strategies employed by each LHD, statistical comparisons to be made across trials, and provides important insight into the types of implementation strategies that could be employed within future efforts to maximise school adoption of effective health promotion programs.

## Supplementary Information

Supplementary Material 1.

## Data Availability

The data presented in this study are available on request from the corresponding author.

## Abbreviations

LHD: Local Health District
NSW: New South Wales
CoP: Community of Practice
NCOIS: National Centre of Implementation Science
HI: High Intensity
BAU: Business as Usual
SES: Socio-Economic Status
ERIC: Expert Recommendations for Implementing Change
ACARA: Australian Curriculum, Assessment and Reporting Authority
